# EGFR and αvβ6 as Promising Targets for Molecular Imaging of Cutaneous and Mucosal Squamous Cell Carcinoma of the Head and Neck Region

**DOI:** 10.3390/cancers12061474

**Published:** 2020-06-05

**Authors:** Victor M. Baart, Chayenne van Duijn, Sylvia L. van Egmond, Willem A. Dijckmeester, Jeroen C. Jansen, Alexander L. Vahrmeijer, Cornelis F. M. Sier, Danielle Cohen

**Affiliations:** 1 Department of Surgery, Leiden University Medical Center, 2333 ZA Leiden, The Netherlands; v.m.baart@lumc.nl (V.M.B.); chayennevanduijn@outlook.com (C.v.D.); a.l.vahrmeijer@lumc.nl (A.L.V.); c.f.m.sier@lumc.nl (C.F.M.S.); 2 Department of Otorhinolaryngology and Head & Neck Surgery, Leiden University Medical Center, 2333 ZA Leiden, The Netherlands; S.L.van_Egmond@lumc.nl (S.L.v.E.); W.A.Dijckmeester@lumc.nl (W.A.D.); J.C.Jansen@lumc.nl (J.C.J.); 3 Department of Pathology, Leiden University Medical Center, 2333 ZA Leiden, The Netherlands

**Keywords:** oral cancer, skin cancer, image-guided surgery, biomarkers

## Abstract

R0 resection is paramount in cutaneous squamous cell carcinoma (CSCC) and head and neck squamous cell carcinoma (HNSCC). However, in the setting of recurrence, immunocompromised patients, or non-keratinizing squamous cell carcinoma (SCC) with a spindle growth pattern, tumor borders are difficult, if not impossible, to determine. Fluorescence-guided surgery (FGS) aids in this differentiation. Potential targets for FGS of CSCC and HNSCC were evaluated. Most sections stained intensely for αvβ6 and epidermal growth factor receptor (EGFR) on tumor cells. Normal epithelium stained less for αvβ6 than for EGFR. In addition, soft tissue and stroma stained negative for both, allowing for clear discrimination of the soft tissue margin. Tumor cells weakly expressed urokinase plasminogen activator receptor (uPAR) while expression on stromal cells was moderate. Normal epithelium rarely expressed uPAR, resulting in clear discrimination of superficial margins. Tumors did not consistently express integrin β3, carcinoembryonic antigen, epithelial cell adhesion molecule, or vascular endothelial growth factor A. In conclusion, αvβ6 and EGFR allowed for precise discrimination of SSC at the surgically problematic soft tissue margins. Superficial margins are ideally distinguished with uPAR. In the future, FGS in the surgically challenging setting of cutaneous and mucosal SCC could benefit from a tailor-made approach, with EGFR and αvβ6 as targets.

## 1. Introduction

Margins are tumor-positive in 6.3–12.8% of tumor resections of cutaneous and mucosal squamous cell carcinomas (SCCs) of the head and neck region [1,2]. Especially in the setting of recurrence after previous R1 resection or irradiation, immunocompromised patients, or non-keratinizing SCC with a spindle growth pattern, tumor borders are difficult, if not impossible, to determine. In these high-risk cases, irradical resection rates can be up to 60% and local recurrence rates as high as 50% [2,3].

Cutaneous squamous cell carcinoma (CSCC) accounts for roughly 20% of all skin malignancies and unlike the most common skin cancer, basal cell carcinoma, has a substantial risk of metastasizing [4]. Furthermore, recurrence rates can exceed 50% in patients with high risk factors, such as head andneck localization, perineural involvement, or immunosuppression [3,5,6,7]. In all these cases, local control by achieving tumor-free margins is paramount in decreasing the risk for metastasis and recurrence [8].

Head and neck squamous cell carcinoma (HNSCC) arises from the mucosal epithelium of the oral cavity, nasal cavity, paranasal sinuses, and pharynx [9]. By incidence, these tumors are the eighth most common cancer types worldwide and account for more than 400,000 deaths annually [10]. Although the last decades have led to significant insights into the risk factors, carcinogenesis, and therapeutic possibilities of HNSCC, the five-year mortality rate is still around a devastating 50% [11]. Considering that incomplete resection rates are currently at 15–30% and are directly associated with poor patient outcomes, a significant gain can be achieved by decreasing positive margin rates [12,13,14].

To reduce the number of positive resection margins, fluorescence-guided imaging (FGS) has been introduced into operating theatres. FGS grants a unique opportunity to visualize tumors and possible (nodal) metastasis using an advanced camera system and fluorescently labelled tracers targeting specific membrane-associated proteins on cancer cells [15]. The proper identification of tumor-specific targets for molecular imaging is key to the success of FGS [16,17]. For HNSCC, epidermal growth factor receptor (EGFR) has been identified as a suitable candidate and various exploratory preclinical and clinical trials have indicated the potential of this concept in aiding surgeons during head and neck tumor removal [18,19]. However, an appropriate study comparing the expression of molecular targets suitable for rapid translation towards the clinic in HNSCC and CSSC for the goal of FGS has not yet been undertaken.

Therefore, this study aims to compare the immunohistochemical expression of EGFR versus αvβ6, integrin β_3_, carcinoembryonic antigen (CEA), epithelial cell adhesion molecule (EpCAM), urokinase plasminogen activator receptor (uPAR), and vascular endothelial growth factor A (VEGF-A) as targets for FGS of high-risk CSCC and HNSCC.

## 2. Results

### 2.1. Patient Characteristics

Tumor tissue from 56 patients, 37 with CSCC and 19 with HNSCC, treated at the Department of Otorhinolaryngology and Head and Neck Surgery in the Leiden University Medical Center (LUMC), were included in the study and stained for the biomarkers. The clinical characteristics of this cohort are included in Table 1. Importantly, 25.0% (14/56) of patients had involved margins and 21.5% (12/56) had narrow margins (<3 mm). Furthermore, 37.8% (14/37) of CSCC patients were immune-compromised, 18.9% (7/37) potentially immune-compromised, and 43.2% (16/37) not immune-compromised. As the compromised patients represent an important group of high-risk cases, a sub-group analysis was performed with the three most promising biomarkers to determine whether immunosuppression altered biomarker expression.

### 2.2. Immunohistochemical Stainings

#### 2.2.1. EGFR

**Figure 1:**
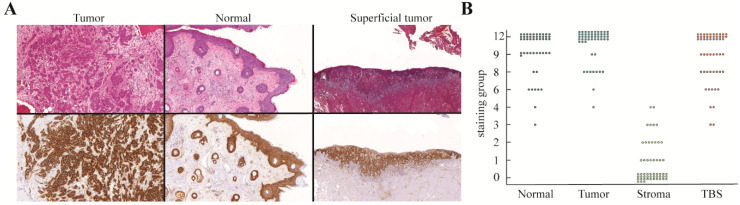
EGFR expression of SCC of the head and neck where (**A**) H&E and EGFR immunohistochemical staining showing the results of a typical tumor (left), normal squamous epithelium and skin adnexa (middle), and a superficial tumor (right). (**B**) Graph demonstrating the distribution of the immunohistochemical staining scores for tumor cells, stromal cells, normal epithelium, and TBS. EGFR: epidermal growth factor receptor, SCC: squamous cell carcinoma, H&E: hematoxylin & eosin, TBS: tumor-border score.

For EGFR, there was intense membranous staining of tumor cells, and a rare tumor also stained weakly in the tumor stroma cell population and subcutaneous tissue. Besides staining within the tumor, normal squamous epithelium and skin adnexa also expressed EGFR with a similar intensity found in the tumor (Figure 1A). This resulted in the following staining scores for tumor cells, stromal cells, and normal epithelium: 12 (12, 12), 0 (0, 1), 12 (9, 12), respectively (Figure 1B).

#### 2.2.2. αvβ6 Integrin

**Figure 2:**
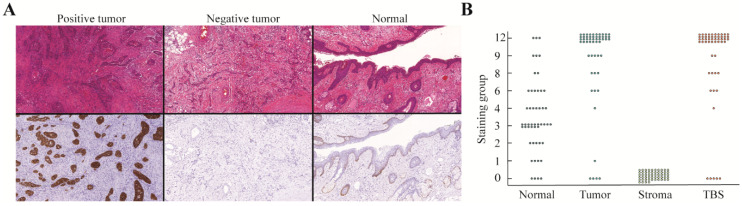
αvβ6 expression of SCC of the head and neck where (**A**) images of H&E and the corresponding αvβ6 immunohistochemical staining showing the results of a positive tumor (left), negative tumor (middle), and normal squamous epithelium. (**B**) Graph demonstrating the distribution of the immunohistochemical staining scores for tumor cells, stromal cells, normal epithelium, and TBS. SCC: squamous cell carcinoma, H&E: hematoxylin & eosin, TBS: tumor-border score.

αvβ6 showed a clear membranous presence and tumor cells were intensely positive with no expression in the tumor stroma. There was varied expression in normal squamous tissue that was mostly restricted to the basal membrane. In well-differentiated tumor areas, only tumor cells of the “pearl-like structures” in contact with the stroma stained positive, leaving the core unstained. Interestingly, an “on/off” phenomenon was seen in CSCC patients, with 13% (*n* = 5) of patients showing no or minimal staining of tumor cells (Figure 2A). Occasionally, muscle tissue showed a weak membranous and cytoplasmic staining. The resulting staining scores for αvβ6 were 12 (9, 12), 0 (0, 0), and 3 (2, 6) for tumor cells, stromal cells, and normal epithelium, respectively (Figure 2B).

#### 2.2.3. uPAR

**Figure 3:**
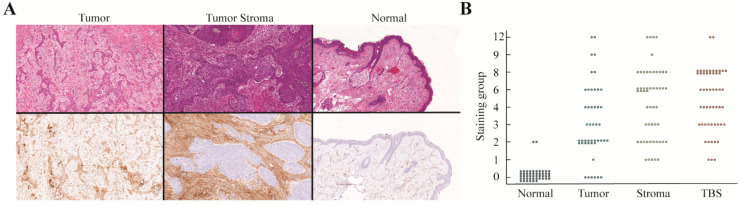
Expression of uPAR of SCC of the head and neck where (**A**) images of H&E and uPAR immunohistochemical staining showing the results of uPAR expression on tumor cells (left), stromal cells (middle), and normal squamous epithelium. (**B**) Graph demonstrating the distribution of the immunohistochemical staining scores for tumor cells, stromal cells, normal epithelium, and TBS. SCC: squamous cell carcinoma, H&E: hematoxylin & eosin, uPAR: urokinase plasminogen activator receptor, TBS: tumor-border score.

Expression of uPAR was seen in most tumors, but with different staining patterns. In 34% (*n* = 18) of tumors more than half of the tumor cells stained with the uPAR antibody, and in 64% (*n* = 34) of cases more than half of the stromal cells stained positive (Figure 3A). Stromal cells expressing uPAR were tumor-associated macrophages, fibroblasts, and neo-angiogenic endothelium found at the invasive margin. Except for two cases, the normal epithelium was consistently negative, as was the surrounding subcutaneous tissue. One (1/53) case with a diffuse immune infiltrate also stained intensely. Median scores were 2 (1, 4), 6 (2, 8), and 0 (0, 0) for tumor, stromal, and normal tissue, respectively (Figure 3B).

#### 2.2.4. VEGF-A

**Figure 4:**
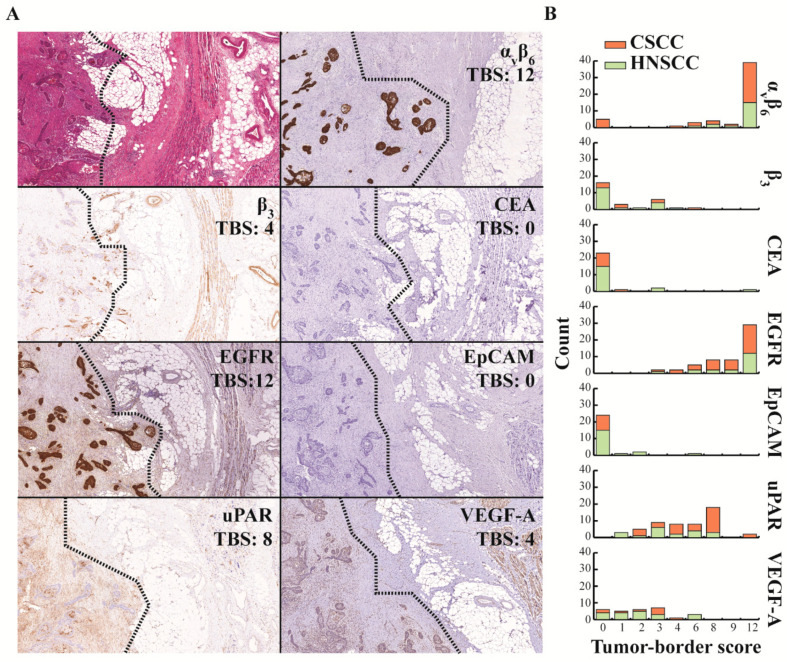
Assessing target expression at the border of SCC using the TBS, where (**A**) images of representative immunohistochemical stainings at 5× magnification from one single case of the border of a SCC with a branching growth pattern. Left of the dotted line is tumor tissue and right is the surrounding tissue. (**B**) TBS categorized by location of the tumor (CSCC vs. HNSCC) for all evaluated targets. TBS: tumor-border score, EGFR: epidermal growth factor receptor, uPAR: urokinase plasminogen activator receptor, CSSC: cutaneous squamous cell carcinoma, HNSCC: head and neck squamous cell carcinoma, CEA: carcinoembryonic antigen, EpCAM: epithelial cell adhesion molecule, VEGF-A: vascular endothelial growth factor-A.

Tumors weakly expressed VEGF-A with antibody staining in both the tumor and the stromal compartment. Abundant VEGF-A expression was also seen regularly in normal squamous epithelium, blood vessels, and muscle tissue, with both a membranous and intracellular presence (Figure 4A). The tumor median staining score was 3 (2, 4), while that of the stromal and healthy tissue was 1 (0, 2) and 2 (1, 3), respectively.

#### 2.2.5. β3 Integrin

Integrin β_3_ expression was mostly absent in tumor cells, except for occasional well-differentiated tumors, where it stained the outer cells weakly. As expected, most of the tumor staining was seen on the endothelium, both inside and outside of the tumor compartment (Figure 4A). This resulted in median staining scores of 0 (0, 2), 3 (2, 3), and 0 (0, 0) for tumor, stromal, and healthy squamous epithelium tissue, respectively.

#### 2.2.6. EpCAM and CEA

EpCAM and CEA were not consistently expressed in tumor, stromal, or normal tissue (Figure 4A). Median EpCAM staining scores were 0 (0, 0) for all three compartments. The median staining scores for CEA were 0 (0, 2), 0 (0, 0), and 0 (0, 0) for tumor, stromal, and normal cells, respectively.

### 2.3. Introducing the Tumor-Border Score (TBS) for the Evaluation of EGFR as a Target for FGS

The appropriateness of a molecular marker for FGS could be semi-quantitatively evaluated by the novel tumor-border score (TBS). By drawing an imaginary line between the tumor and surrounding normal tissue and comparing the percentage and intensity of cell staining, the TBS compares the tumor and the surrounding tissue expression across all margins, whether these are mucosal or soft tissue (Figure S1). The TBS method was assessed using EGFR because its utility has already been demonstrated in clinical trials. The median TBS was 12 (8, 12) for all tumors (*n* = 54) and did not differ, particularly between CSCC and HNSCC (Figure 4B). As both tumor cells and healthy squamous epithelium tissue scored high for EGFR, superficial tumors with mostly superficial margins resulted in a relatively low TBS.

### 2.4. TBS of the Other Molecular Targets

Figure 4A shows images of a representative case of SCC from the head and neck region stained for all seven evaluated targets and with their respective TBS. Integrin β_3_, CEA, and EpCAM were not suitable targets for FGS, with TBSs 0 (0, 3), 0 (0, 0), and 0 (0, 0), respectively, as indicated in Figure 4B. VEGF-A presented a low TBS with a median score of 2 (1, 3), as expression was also seen in normal epithelium, endothelium, and muscle tissue. A moderate TBS was achieved with the uPAR staining, resulting in a median score of 6 (3, 8), mostly because, although uPAR expression was present, it rarely stained intensely. Lastly, αvβ6 integrin resulted in the highest median TBS of 12 (8, 12), even though 11% (*n* = 6) cases did not stain positive in the tumor cells, resulting in a TBS of 0 for these cases (Figure 4B).

### 2.5. Target Expression in Immune-Compromised Patients

Patients with an immune-compromised status inherently have a higher risk of developing cutaneous squamous cell carcinomas [20,21]. On top of the increased incidence, these tumors have a more insidious course of disease, justifying the need for fluorescence-guided resections [22]. Whether the same molecular targets could be used for this subset of CSCC patients was assessed by using the results of the candidates that proved usable by the TBSs, i.e., EGFR, αvβ6, and uPAR. There was a significant difference in tumor αvβ6 expression between immune statuses, χ*^2^* (2) = 6.362, *p* = 0.042, with a mean rank score of 14.11 for immune-compromised, 22.46 for competent, and 16.86 for possibly compromised patients. Post hoc testing provided evidence that there was a significant difference between the immune-compromised and competent patients (*p* = 0.038, adjusted using the Bonferroni correction). The other pairs revealed no significant difference. There were no differences for uPAR and EGFR across immune statuses.

## 3. Discussion

Considering that incomplete resection rates of high-risk CSCC and HNSCC are currently as high as 60%, and are directly associated with poor patient outcomes, finding methods to decrease positive margins is of vital importance. FGS with targeted fluorescent tracers offers a unique opportunity to provide real-time visual feedback on the location of the resection margins and the possible presence of metastasis, without altering the view of the operative field [15]. However, the successful application of fluorescence imaging is crucial for the selection of appropriate tracers [23]. Ideal tracers will target cell membrane-associated proteins that are overexpressed in cancerous, and absent in non-cancerous, tissue.

With these characteristics in mind, we evaluated seven molecular imaging tracers that are currently in various stages of clinical translation for their potential as suitable molecular targets for FGS of SSC of the head and neck region. Our results show that EGFR, αvβ6, and uPAR are promising targets. Importantly, our data, including a wide variety of patients and settings, underline that a one size fits all approach is not feasible: EGFR allowed clear delineation between CSCC or HNSCC and the surrounding tissue, except in areas where normal squamous epithelium, glands, and adnexa were in proximity to the tumor, and αvβ6 showed intense tumor expression with minimal staining in the basal layer of the dermis, but also exhibited an “on/off” phenomenon [24,25,26,27,28,29,30]. Lastly, uPAR showed a tumor-specific heterogeneous staining pattern in both tumor and stromal cells [23,30,31,32].

**Figure 5:**
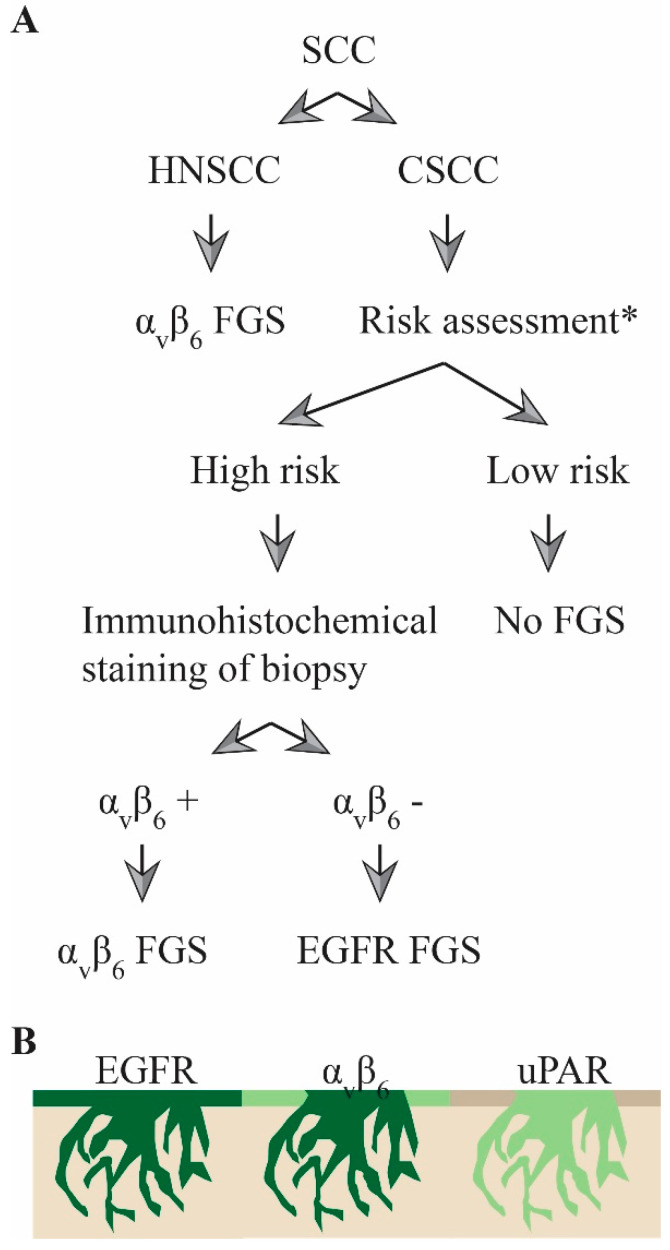
Three-tiered approach for FGS of SCC where (**A**) proposed algorithm to decide what target to use during FGS of squamous cell carcinoma of the head and neck region. (**B**) Illustrations depicting, based on the immunohistochemical results, where fluorescence would be expected during FGS using EGFR-, αvβ6-, or uPAR-based probes. Dark green represents more fluorescence than light green. FGS: fluorescence-guided surgery, HNSCC: head and neck squamous cell carcinoma, CSCC: cutaneous squamous cell carcinoma. * as determined by the NCCN or AJCC criteria for high-risk CSCC.

Considering these results, in the future, a three-tiered approach can be visualized to determine whether FGS is indicated and what tracer should ideally be used (Figure 5A). Initially, HNSCC and CSCC should be differentiated. For HNSCC, αvβ6 is preferred over EGFR due to its lower expression in normal squamous epithelium. For cutaneous lesions, a further distinction should be made between cases of high and low metastatic risk. With low-risk tumors, FGS is not mandatory, while the biopsies of high-risk patients should be stained immunohistochemically for αvβ6, after which the most appropriate tracer can be used. As expression was homogenously positive in the whole tumor for both markers, false positives or false negatives in tumor biopsies due to tumor heterogeneity should not be a problem. In αvβ6-negative cases, where superficial margins are possibly tumor-positive, surgeons can opt for uPAR-targeting tracers (Figure 5B).

The expression of EGFR in normal squamous epithelium could lead to the aggregation of tracer and subsequent fluorescence in the mucosa or skin. To circumvent this effect, preloading with unlabeled tracer has been performed in oral cancer clinical trials evaluating cetuximab- and panitumumab-based FGS [18,33,34]. However, recent studies have shown that off-target fluorescence still occurred after preloading and no difference in tumor-to-background ratios and mean fluorescent intensities between no loading and preloading cohorts exist [35,36]. Consequently, the expression of EGFR in the normal squamous epithelium is a limiting factor, especially in superficial growing tumors.

Our data showed a puzzling disadvantage of αvβ6 as a target for FGS of CSCC, because of an “on/off” phenomenon in the immunohistochemical staining. In 13% of cases, immunohistochemical staining was completely negative. A compromised immune status seemed to be associated with lower αvβ6 tumor expression. This is important, as immune-suppressed patients represent a high-risk group for aggressive tumors and consequently challenging resections [20,21,22]. An explanation for the “on/off” phenomenon remains to be elucidated. Mechanistically, αvβ6 has been implicated in tumor genesis as a direct upstream regulator of matrix metalloproteinases and transforming growth factor-β (TGF-β), where the latter plays a vital role in the immune evasion of cancer cells [37,38]. Theoretically, one could speculate that immune evasion is not an essential hallmark of cancer in immune-compromised patients, and consequently αvβ6 regulating TGF-β loses its significance in tumor genesis. Nonetheless, whether our observations in a small cohort of patients and the pathway-related mechanisms are essential for specific subgroups of patients should be tested and confirmed in larger groups. While fluorescence-based clinical studies are currently being set up, an early Positron emission tomography–computed tomography (PET-CT) study demonstrated that the αvβ6-targeting tracer ^68^Ga-DOTA-SFITGv6 was more specific than ^18^F-FDG for the detection of cancerous lesions [39].

A disadvantage of uPAR encountered in this study was the intensity of the immunohistochemical staining for uPAR, which was considerably less than for EGFR and αvβ6. This can probably be explained by the relatively low copy numbers of uPAR per cell, even if more cells than the malignant tumor cells are targeted [40]. Furthermore, the low intensity might be a drawback of using the immunohistochemical staining technique and this might not be an issue for in vivo imaging. In fact, first-in-human clinical trials with the uPAR AE-105 PET tracer have demonstrated the capability to identify primary and metastatic lesions of various tumor types and currently seven clinical trials, including one with HNSCC, are running to further assess the potential of uPAR-imaging [41,42]. Regarding fluorescence molecular imaging, various groups have published advanced preclinical studies, and clinical trials should be following soon [31,43]. Ultimately, the advantage of performing fluorescent guided surgery with a uPAR-targeting tracer, as opposed to EGFR or αvβ6, is the non-existent expression in normal tissue and the uPAR expression in stromal cells. Therefore, performing FGS with a uPAR-targeting tracer will automatically also result in fluorescent stromal cells, and consequently the removal of stroma by the surgeon.

The limitations of this study include the semi-quantitative evaluation of the targets and their comparison. However, these are inherent to immunohistochemical methods [44]. The choice of primary antibodies is pivotal. In this study, only antibodies that interacted with extracellular epitopes close to binding domain of the clinical tracers were used. Although clinical trials will need to confirm the binding characteristics of the appropriate tracers, these antibodies give a fair indication whether the extracellular domain of the target is present. Interpretation is further limited by the small sample size, especially for subgroup analyses. However, even with large cohorts and validated antibodies, staining results can vary depending on the representative tumor specimen and scoring method chosen [45].

For this study, the novel scoring method, TBS, was introduced, adapted to the purpose of evaluating targets for FGS. The TBS, using specimens that contain both tumor and surrounding tissue, evaluates the staining difference between the tumor border and the surrounding tissue, allowing the precise evaluation of whether a target is suitable for FGS. Often the expression between tumor and healthy cells is compared by comparing the tumor staining with its healthy counterpart and not the normal tissue surrounding the tumor. However, this does not account for the expression of the markers in the surgically more troublesome soft tissue margins [46]. Another scoring method to evaluate markers for molecular imaging that has been used in the literature is the Target Selection Criteria (TASC) scoring system. In this score, targets are scored based on seven characteristics. However, the importance of certain criteria of the TASC score, for example, the internalization of the probe, are questionable, while other criteria, such as a T/N of greater than 10, are challenging to measure [47]. All in all, the TBS allows an alternative assessment for the suitability of a marker for FGS.

## 4. Materials and Methods

### 4.1. Patient and Tissue Selection

The medical records of patients who underwent surgical resection for confirmed squamous cell carcinoma at the department of Otorhinolaryngology and Head and Neck Surgery of the LUMC between January 2014 and February 2019 were retrospectively reviewed. Patients were sub-grouped based on tumor localization (CSCC *n* = 37, HNSCC *n* = 19). Clinicopathological data were collected to assess the immune status of the patients. Patients with a positive history for an organ transplant at least one year before tumor occurrence and subsequent use of immune-suppressive medication were considered immune-compromised. Patients who did not have a transplant history but used immune-suppressive medication in the year before their tumor-associated surgery were regarded as possibly immune-compromised. Immune-competent patients had no history of transplant or immune-suppressive drug use.

Tissue samples were selected based on the simultaneous presence of tumor tissue, surrounding unaffected tissue, and pre-existent normal squamous epithelium. A specialized, experienced pathologist (D.C.) reviewed the tissue samples before their inclusion in the study. The local ethics review board (Medische-Ethische Toetsingscommissie Leiden Den Haag Delft (METC-LDD)) approved the study protocol and research was conducted according to Code Goed Gebruik (Human Tissue and Medical Research: Code of conduct for responsible use (2011)) and Code Goed Gedrag (Code of Conduct for Medical Research (2004)). Both codes are prescribed by the Dutch Federation of Medical Scientific Societies. Informed consent was not needed for this study. Samples and data were non-identifiable and used in accordance with the 1964 Helsinki declaration.

### 4.2. Antibodies and Reagents

The molecular target selection was based on both the potential of a quick clinical translation (EGFR, CEA, EpCAM, VEGF-A) and the potential specificity for squamous cell carcinoma (αvβ6, integrin β_3_ and uPAR). The antibodies and reagents used for the immunohistochemical staining can be found in Table S1.

### 4.3. Immunohistochemistry

Formalin-fixed, paraffin-embedded tissue blocks from the department of Pathology of the LUMC were collected and sliced into tissue sections of 4 µm. Sections were deparaffinized in xylene and rehydrated via serially diluted ethanol solutions. Endogenous peroxide was blocked for 20 min with 0.3% hydrogen peroxide diluted in demi water. When appropriate, antigen retrieval was performed as described in Table S1. Subsequently, sections were incubated overnight at room temperature with the primary antibody. The optimal dilution for each of the primary antibodies was determined beforehand on squamous cell tissue (see Table S1). Slides were washed three times with phosphate-buffered saline (pH = 7.5) before incubating the slides for 30 min at room temperature with the secondary antibody, followed by another washing step. Staining was visualized with 3,3-diaminobenzidine tetrahydrochloride solution (K3468, Agilent Technologies, Inc., Santa Clara, CA, USA) for 5 min at room temperature and counterstained for 20 s with hematoxylin (4085.9002, VWR International, Amsterdam, The Netherlands). After the dehydration of the slides, they were mounted in Pertex (0081EX, Histolab, Askim, Sweden).

### 4.4. Immunohistochemistry Analysis

Stained sections were digitalized with the Panoramic Digital Slide Scanner and viewed with CaseViewer 2.3 (both from 3D Histech, Budapest, Hungary). The evaluation of the immunohistochemical staining of all tissues occurred independently by two observers after a training period by an experienced pathologist. Upon disagreement, observers discussed together to reach a consensus. If no agreement could be reached, the pathologist determined the final score. The expression of each molecular biomarker was assessed for its presence on tumor, stromal, and normal squamous epithelial cells based on an intensity and percentage score. The intensity was subdivided into four groups (0 = none, 1 = weak, 2 = moderate, 3 = intense) and the percentage of cells in five groups (0 = 0–5%, 1 = 6–25%, 2 = 26–50%, 3 = 51–75%, 4 > 75%). The final intensity and percentage scores were multiplied together to get a total score, resulting in a nine-point ordinal scale (0, 1, 2, 3, 4, 6, 8, 9, 12).

Whether the biomarker was suitable as a molecular tumor-imaging target was assessed by the newly introduced tumor-border score (TBS). The difference in the expression of the biomarker between cancerous and non-cancerous tissue is relevant for tumor imaging, whether that be normal epithelium, subcutaneous tissue, or other soft tissue [23]. For the TBS, an imaginary line was drawn on the hematoxylin & eosin (H&E)-stained slide along the tumor border by the pathologist, and the difference in intensity between the tumor area and non-cancerous tissue (0 = no difference, 1 = slight difference, 2 = moderate difference, 3 = large difference) and the percentage of border that contained this difference (0 = 0–5%, 1 = 6–25%, 2 = 26–50%, 3 = 51–75%, 4 > 75%) was scored. These scores were multiplied, resulting in a nine-point ordinal scale (0, 1, 2, 3, 4, 6, 8, 9, 12), indicating the usefulness of the molecular target for tumor-imaging. Figure S1 contains examples.

### 4.5. Statistical Analysis

Statistical analysis was performed using IBM SPSS Statistics 23.0 (SPSS, IBM Corporation, Armonk, NY, USA). The results were reported as medians, followed by the 1st and 3rd quartile in brackets. The Kruskal–Wallis one-way ANOVA test with Dunn’s post hoc test and Bonferroni correction determined the difference in staining between patients with various immune statuses. Results of *p* < 0.05 were considered statistically significant.

## 5. Conclusions

In conclusion, αvβ6 and EGFR allowed for the precise discrimination of SSC at the often more problematic soft tissue margins in CSCC and HNSCC. When superficial margins are at risk for irradical resection due to difficult clinical tumor delineation, uPAR is a promising target. In the future, FGS in the surgically challenging setting of high-risk CSCC and HNSCC could benefit from a tailor-made approach, with EGFR and αvβ6 as promising targets.

## Figures and Tables

**Table 1:** Characteristics of high-risk SCC patients subdivided by origin: CSCC vs. HNSCC. SCC: squamous cell carcinoma, CSCC: cutaneous squamous cell carcinoma, HNSCC: head and neck squamous cell carcinoma, SD: standard deviation, n.a.: not applicable.

Characteristics	Total Population (*n* = 56)	CSCC (*n* = 37)	HNSCC (*n* = 19)
Age, mean (SD)	70 (11)	72 (10)	67 (11)
Male gender, *n* (%)	49 (87.5%)	34 (91.9%)	15 (78.9%)
Tumor differentiation, *n* (%)			
Well differentiated	4 (7.1%)	3 (8.1%)	1 (5.3%)
Moderately differentiated	18 (32.1%)	8 (21.6%)	10 (52.6%)
Poorly differentiated	10 (17.9%)	8 (21.6%)	2 (10.5%)
Missing	24 (42.9%)	18 (48.6%)	6 (31.6%)
Primary tumor, *n* (%)			
pT1	31 (55.3%)	22 (59.5%)	9 (47.4)
pT2	11 (19.6%)	10 (27.0%)	1 (5.3%)
pT3	4 (7.1%)	2 (5.4%)	2 (10.5%)
pT4	10 (17.9%)	3 (8.1%)	7 (36.8%)
Regional lymph nodes, *n* (%)			
cN0, pN not assessed	41 (73.2%)	32 (86.5%)	9 (47.4%)
pN0	8 (14.3%)	1 (2.7%)	7 (36.8%)
pN1	2 (3.6%)	1 (2.7%)	1 (5.3%)
pN2	5 (9.0%)	3 (8.1%)	2 (10.5%)
Surgical margin status, *n* (%)			
R0	30 (53.6%)	19 (51.4%)	11 (57.9%)
Narrow	12 (21.4%)	7 (18.9%)	5 (26.3%)
R1	14 (25.0%)	11 (29.7%)	3 (15.8%)
Immune Status, *n* (%)			
Compromised	n.a.	14 (37.8%)	n.a.
Potentially compromised	n.a.	7 (18.9%)	n.a.
Not compromised	n.a.	16 (43.2%)	n.a.

## Supplementary Materials

The following are available online at https://www.mdpi.com/2072-6694/12/6/1474/s1, Figure S1: Evaluating the suitability of Targets for FGS, Table S1: Antibodies and reagents used.
