# Supplementary material for: EGFR and αvβ6 as Promising Targets for Molecular Imaging of Cutaneous and Mucosal Squamous Cell Carcinoma of the Head and Neck Region
Source: Cancers (Basel). 2020 Jun 5;12(6):1474. doi: 10.3390/cancers12061474 (PMC7352159; doi:10.3390/cancers12061474)
Supplement: Supplementary file 1 [file cancers-12-01474-s001.pdf]

cancers

MDPI

Article

EGFR and αvβ6 as Promising Targets for Molecular Imaging of Cutaneous and Mucosal Squamous Cell Carcinoma of the Head and Neck Region

Victor M. Baart, Chayenne van Duijn, Sylvia L. van Egmond, Willem A. Dijckmeester, Jeroen C. Jansen, Alexander L. Vahrmeijer, Cornelis F. M. Sier and Danielle Cohen

Supplementary Materials

Table S1. Antibodies and reagents used.

Target	Catalog Number	Source	Species	Monoclonal/ Polyclonal	Antigen Retrieval	Dilution (μg/mL)
			Primary Antibodies			
αvβ6	6.2A1	Biogen, Inc., Cambridge, MA, USA	Mouse	Monoclonal	0.4% pepsin (S3002 Agilent) 37 °C for 10 min.	0.5
β3	#13166	Cell Signaling Technology, Inc., Danvers, MA, USA	Mouse	Monoclonal	Target retrieval solution, low pH (K8005 Agilent) 95 °C for 10 min with PT Link (Agilent).	100
CEA	SC-23928	Santa Cruz Biotechnology, Inc., Dallas, TX, USA	Mouse	Monoclonal	Target retrieval solution, low pH (K8005 Agilent) 95 °C for 10 min with PT Link (Agilent).	0.2
EGFR	M7239	Agilent Technologies, Inc., Santa Clara, CA, USA	Mouse	Monoclonal	0.4% pepsin (S3002 Agilent) 37 °C for 10 min.	1.4
EpCAM	MA5-12436	Thermo Fisher Scientific, Inc., Waltham, MA, USA	Mouse	Monoclonal	0.1% trypsin (T7409 Sigma Aldrich) 37° C for 30 min.	0.3
uPAR	ATN617	Kind gift of A.P. Mazar	Mouse	Monoclonal	Target retrieval solution, low pH (K8005 Agilent) 95 °C for 10 min with PT Link (Agilent).	1.2
VEGF-A	RB-9031-P0-A	Thermo Fisher Scientific, Inc., Waltham, MA, USA	Rabbit	Polyclonal	Target retrieval solution, low pH (K8005 Agilent) 95 °C for 10 min with PT Link (Agilent).	0.3

Table S1. *Cont.*

Secondary Antibodies						
anti-mouse	K4001	Agilent Technologies, Inc., Santa Clara, CA, USA	–	–	–	Ready-to-use
anti-rabbit	K4003	Agilent Technologies, Inc., Santa Clara, CA, USA	–	–	–	Ready-to-use

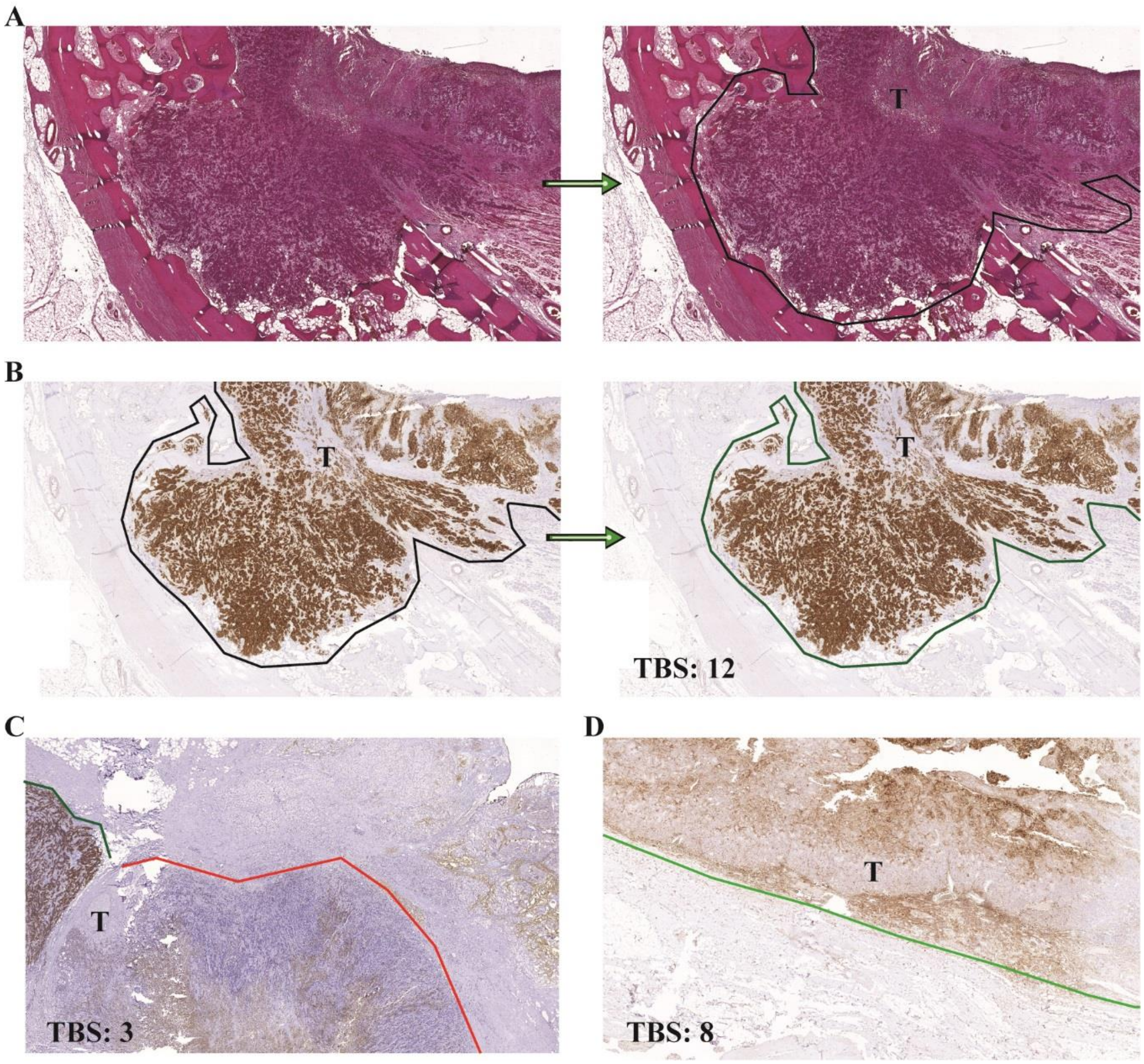

Figure S1. Evaluating the suitability of targets for FGS by the new TBS method is done as described: (**A**) A pathologist marked the tumour borders on H&E stainings. (**B**) These borders were evaluated using immunohistochemical staining for the difference in intensity between the tumour area and surrounding tissue and the percentage of the border that stained with this intensity difference. The TBS is a product of the intensity and the group number the percentage score fits in (0 < 5%, 1 = 6–25%, 2 = 26–50%, 3 = 51–75%, 4 > 75%). In this case the intensity difference is 3 and the percentage group is 4 (>75% of the border stains with this percentage difference) resulting in a TBS of 12. (**C**) In this case the intensity difference is 3 but less than 25% of the border stains with this difference (percentage group 1), resulting in a TBS of 3. (**D**) The TBS method does not discriminate between tumour or stroma cell staining. In this case, the tumour staining is weak, but the stroma staining along the border still allows for the differentiation between tumour and normal tissue. The intensity difference is 2, and the percentage group is 4, resulting in a TBS of 8. All images are taken at 2× magnification. Black line: border between tumour and surrounding tissue, dark green line: intensity difference of 3, light green line: intensity difference of 2, red line: intensity difference of 0. T: tumour, TBS: tumour-border score, FGS: molecular fluorescence-guided surgery, H&E: hematoxylin & eosin staining.
